# Comparison of the results of stop-start technique with stop-start technique and sphincter control training applied in premature ejaculation treatment

**DOI:** 10.1371/journal.pone.0283091

**Published:** 2023-08-10

**Authors:** Kazım Doğan, Cem Keçe

**Affiliations:** 1 Urologist, Istinye University, Istanbul, Turkey; 2 Psychotherapist, President of IICPI, Ankara, Turkey; Sant Anna Hospital: Clinica Sant’Anna, SWITZERLAND

## Abstract

**Background:**

The aim of this study is to compare the results of stop-start technique with stop-start technique together with sphincter control training applied in the treatment of premature ejaculation.

**Methods:**

This research was conducted as a pre-test post-test quasi-experimental study. The sample of the study consisted of 80 men. The study was conducted on men who applied to the urology outpatient clinic of LIV Hospital, a prıvate hospital, in Gaziantep, Turkey, between 01 October 2021 and 01 March 2022. “Personal Information Form”, “Intravaginal Ejaculation Latency Time (IELT)”, “Fold Increase Intravaginal Ejaculation Latency Time (F-IELT)” “Premature Ejaculation Diagnostic Tool (PEDT) Questionnaire” and “Arabic Index Premature Ejaculation (AIPE)” were used as the data collection tools. Behavioral therapy, consisting of a total of 6 sessions, was applied once every two weeks, with each session lasting for 45 minutes. After 3rd and 6th months from the beginning of the application, the data collection tools were applied again. “Stop-Start Technique (Group A)” and “Stop-Start Technique and Sphincter Control Training (Group B)” were used in the treatment.

**Results:**

In both groups, the IELT and AIPE values after 3rd and 6th months from the beginning of the application were statistically higher than those obtained before (p<0.05). IELT and AIPE values increased more in Group B than Group A (p<0.05). F-IELT values after 6th months from the beginning of the application were found to be statistically significant with a low level of effect size than those obtained before (p<0.05, Cohen’s d = 0.027). In both groups, the PEDT values in the 3rd and 6th months after the application were statistically lower than those seen before (p<0.05). PEDT value decreased more in Group B than Group A (p<0.05). The differences between the two groups’ IELT (Cohen’s d = 0.011), AIPE (Cohen’s d = 0.044), and PEDT (Cohen’s d = 0.066) values in the 3rd month after the application and IELT (Cohen’s d = 0.025), AIPE (Cohen’s d = 0.048), and PEDT (Cohen’s d = 0.024) values in the 6th month after the application were found to be clinically weak.

**Conclusions:**

It was determined that the stop-start technique given to men with premature ejaculation increased the time spent in the vagina and eliminated the problem of premature ejaculation. It was determined that the stop-start technique in combination with sphincter control training were more effective than the stop-start technique alone.

## Introduction

Premature Ejaculation (PE) is defined as a pattern of ejaculation during sexual activity with a partner, continuously or recurrently, before, as soon as or within about one minute after entering the vagina, and before the person’s wish [1]. The definition of PE made by the International Society for Sexual Medicine (ISSM) in 2014 is divided into two subtypes. Lifelong Premature Ejaculation (L-PE) is a condition (i) in which ejaculation always or almost always occurs before the vaginal penetration or within 1 minute after the vaginal penetration, (ii) in which inability to delay ejaculation is experienced and (iii) in which frustration, sadness, mental distress and sexual avoidance issues are felt and experienced by the individuals as a result of this problem. The other type, acquired premature ejaculation (A-PE), differs from lifelong PE by the onset of PE complaints in individuals with previously normal ejaculation performance and their ejaculation occurring within about 3 minutes [2]. Premature ejaculation is one of the most common male sexual problems with a rate of 26.2% worldwide [3]. The prevalence of premature ejaculation in Turkey was determined to be 20% [4]. Premature ejaculation has many negative psychological effects such as decreased self-esteem, performance anxiety and interpersonal conflict in men [5]. In the treatment of premature ejaculation, various systemic drugs such as selective serotonin reuptake inhibitors (SSRI), tricyclic antidepressants, 5 phosphodiesterase inhibitors (5FDEI) as well as local anesthetic creams and sprays are applied [6, 7]. Although SSRIs are widely used, patient compliance is not very good due to their side effects and treatment dropout rates are high [8, 9].

In addition to all pharmacological treatments, we know that non-pharmacological treatments such as behavioral treatments, psychotherapy and pelvic floor exercises are used in the treatment of premature ejaculation. Behavioral therapy and psychotherapy have two main goals. The first of these includes sexual counseling aimed at resolving psychological and interpersonal conflicts that may cause premature ejaculation. Secondly, it includes behavioral treatment to improve men’s sexual skills, delay ejaculation and increase their sexual self-confidence [10]. The two commonly used behavioral methods are the stop-start and the squeeze technique [11]. The stop-start technique was first described by Semans (1956). In this technique, the man or partner stimulates the penis until the feeling of ejaculation arises, then stops the stimulation and waits until this feeling fades away. This technique is repeated several times until ejaculation [12]. The squeeze technique was defined by Masters and Johnson (1970) [13]. In this technique, the man or his partner gives a warning to the penis until the feeling of ejaculation comes, then the glans penis is squeezed, and the feeling of ejaculation is expected to fade away. This technique is repeated several times until ejaculation [13, 14]. When the literature is examined, many studies have revealed the positive effect of the stop-start technique on premature ejaculation [15, 16]. For example, in the study of Makwana and Patil (2012), it was stated that the stop-start technique applied to 30 men increased the time spent in the vagina [15]. In addition, in the study of Pavone et al. (2017), the stop-start technique was found to improve the symptoms of premature ejaculation [16]. In addition to all these classical behavioral approaches and the technique of stop-start in the treatment of premature ejaculation in the literature, there is also a study conducted with the support of a medical device to assist masturbation [17, 18].

Another technique is pelvic floor exercises. There are many studies showing that pelvic floor exercises can be used in the treatment of premature ejaculation [19, 20]. The main goal of these exercises is to increase awareness on the anal sphincter and external urethral sphincter and to gain the ability to keep it relaxed. In this way, it has been shown to have significant benefits on ejaculation control [21]. The absence of any detected side effects and the fact that the 36-month results are still effective suggest that there is no obstacle to the widespread use of this method [21, 22]. In the literature, Jiang et al. (2020) and Rodríguez et al. (2019) found that sphincter control has positive effects on premature ejaculation [19, 20].

This study aimed to compare the results of the stop-start technique with the stop-start technique applied in conjunction with sphincter control training in the treatment of premature ejaculation.

### Research hypotheses

H_0_: Stop-start technique or stop-start technique together with sphincter control training have no effect on Intravaginal Ejaculation Latency Time (IELT), Fold Increase Intravaginal Ejaculation Latency Time (F-IELT), Premature Ejaculation Diagnostic Tool (PEDT) and Arabic Index Premature Ejaculation (AIPE) scores.
H_1_: Applying the stop-start technique increases the IELT and F-IELT score.
H_2_: Applying the stop-start technique reduces the PEDT score.
H_3_: Applying the stop-start technique increases the AIPE score.
H_4_: The stop-start technique together with the sphincter control training increases the IELT and F-IELT score.
H_5_: The stop-start technique together with the sphincter control training reduces the PEDT score.
H_6_: The stop-start technique together with the sphincter control training increases the AIPE score.
H_7_: The stop-start technique together with the sphincter control training is more effective than the stop-start technique alone.

## Materials and methods

### Research design

The research was conducted as a pre-test post-test quasi-experimental study to compare the results of stop-start technique with the results of the stop-start technique together with sphincter control training applied in the treatment of premature ejaculation. The study was conducted on men who applied to the urology outpatient clinic of LIV Hospital, a prıvate hospital, in Gaziantep, Turkey, between 01 October 2021 and 01 March 2022. There are 21 polyclinics and 1 emergency service in the hospital where the research was conducted.

### Population and sample of the research

**Fig 1:**
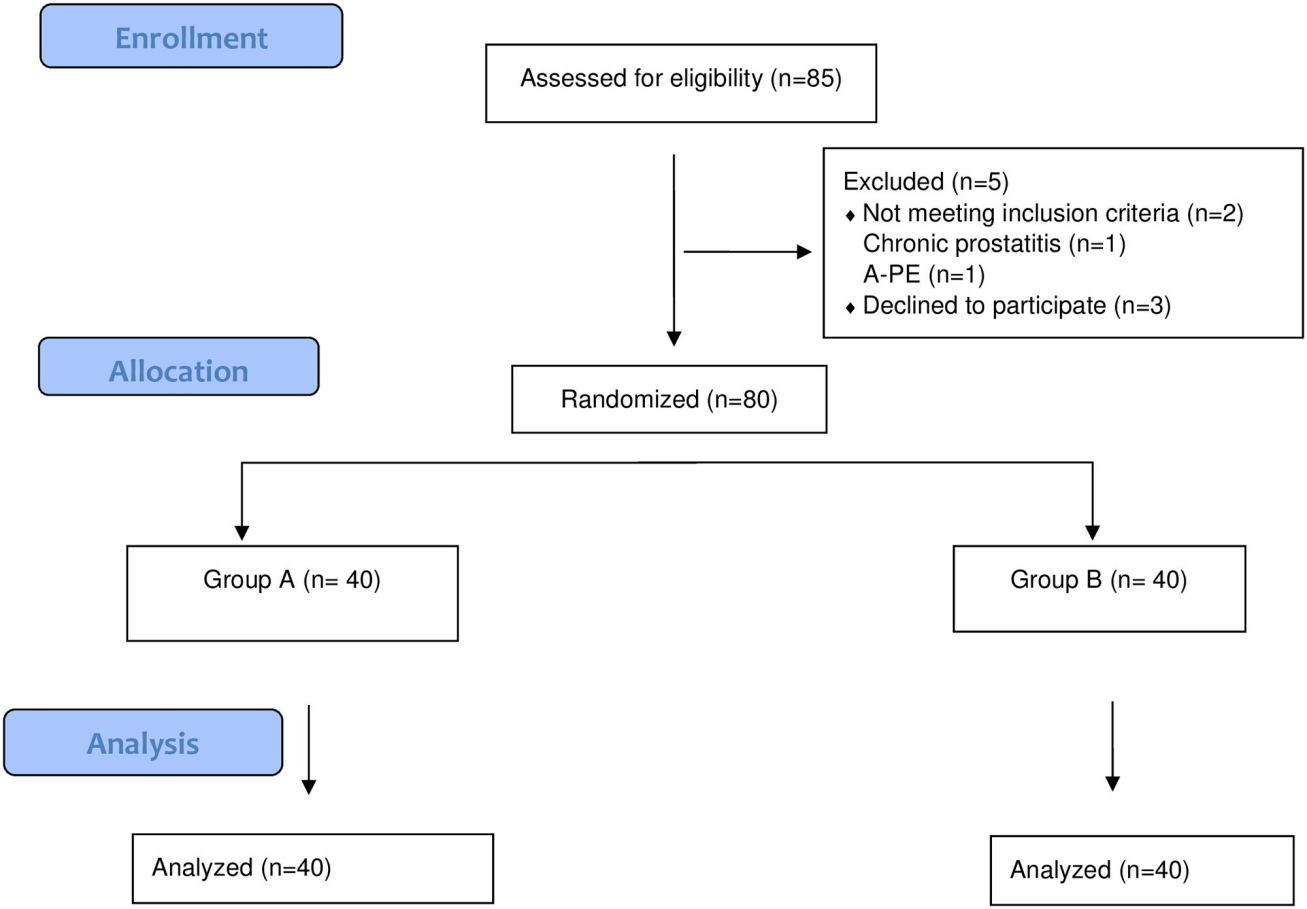
Flow plan of the men participating in the study (Consort).

The population of the study consisted of all men who applied to the LIV Hospital urology outpatient clinic with the diagnosis of premature ejaculation. In the calculation of the sample of the study, a literature review was made, and power analysis was used [19]. The sample size was calculated using the G-Power version 3.9.1 program. The minimum number of males required for each group was found to be 23 (α = 0.05, 1-β = 0.80) for the expectation that there would be a significant difference in the pre-test and post-test evaluation at a large effect size level (Cohen’s d = 0.5) in terms of IELT in the study group. However, to strengthen and generalize the research results, 40 men were included in each study group. During the research, 85 men were reached. 2 men were not included in the study because they did not meet the inclusion criteria, and 3 men gave up during the implementation of the study. As a result, the sample of the study consisted of 80 men (Fig 1).

#### Study inclusion criteria

1. Men who volunteered to participate in the study
2. Men who had no vision, hearing, or speech problems
3. Men who were literate
4. Men aged between 18 and 65
5. Men diagnosed with premature ejaculation according to DSM-5 criteria
6. Men with L-PE
7. Men without any medical disease (psychiatric diseases, pelvic anatomical disorder, urinary system infection, chronic prostatitis, etc.) that might cause PE
8. Men whose IELT duration lasted for less than 1 minute
9. Men whose PEDT scores were above 11
10. Men who had an uninterrupted heterosexual relationship for the past 6 months.

#### Exclusion criteria of volunteers from the study

1. Men who were illiterate
2. Men who did not give a consent for the interviews
3. Men with a medical disease (psychiatric diseases, pelvic anatomical disorder, urinary system infection, chronic prostatitis, etc.) that might cause PE
4. Men who, in addition to premature ejaculation, had other sexual dysfunctions (erectile dysfunction, sexual reluctance, etc.)
5. Men whose IELT duration lasted over 1 minute
6. Men whose PEDT scores were below 11
7. Men with A-PE
8. Men who were having a homosexual relationship
9. Men who did not have an uninterrupted sexual relationship for the past 6 months

#### Randomization

Medcalc version 18.11.3 was used for the randomization list for the assignment of men to the groups. Experimental groups were divided into two groups as “stop-start technique (Group A)” and “stop-start technique together with sphincter control training (Group B)” group. Men diagnosed with premature ejaculation in the clinic where the study was conducted were not kept waiting without treatment, and a control group was not formed. In our study, a control group was not formed because it would not be ethical to monitor a patient with a medical problem without making any intervention.

#### Ethics

Written permission to conduct the study was obtained from the Istinye University Clinical Research Ethics Committee (August 23, 2021-Issue 21–67) and the hospital management. Written and verbal consents were obtained from the participants, who were informed that their participation in the study was completely voluntary and that they could withdraw from the study whenever they wanted. After verbal explanations were given to the participants, written informed consent forms were signed by them. There were no underage participants in the present study. This research was conducted in accordance with the provisions of the Declaration of Helsinki, revised in Brazil in 2013.

### Data collection

#### Preparation of data collection tools

In this study, data collection tools were prepared by the researcher by examining the literature on the subject [19, 23]. Data collection tools consist of three parts:

*Personal Information Form*. Includes questions about the individual’s socio-demographic characteristics and premature ejaculation status (age, marital status, education level, the time of being diagnosed with premature ejaculation).

*Intravaginal Ejaculation Latency Time (IELT)*. Developed by Waldinger et al. (1994) to standardize the definition of premature ejaculation [24]. In this definition, the time from vaginal penetration to ejaculation is measured with a chronometer. The fold increase (F-IELT) of the IELT is a measure of the extent of the ejaculation delay. The F-IELT is calculated by dividing the geometric mean IELT after treatment by the geometric mean IELT at the beginning of treatment [25].

*Premature Ejaculation Diagnostic Tool (PEDT)*. Developed by Symonds et al. (2007) to better define premature ejaculation for use in clinical studies, this is a 5-point Likert-type scale consisting of 5 items [26]. The scale was adapted to Turkish by Serefoglu et al. (2009). The highest score that can be obtained from the scale is 20.0 and the lowest score is 0.0. Scores equal or higher than 11 are defined as “PE”; scores of 9–10 are defined as “possible PE”; and scores of eight or less are defined as “no PE”. The validity and reliability study of the scale found the Cronbach alpha reliability coefficient to be 0.75 [27]. The internal consistency analysis for the scale found the Cronbach alpha reliability coefficient to be 0.71.

*Arabic Index Premature Ejaculation (AIPE)*. Developed by Arafa and Shamloul in 2007, the scale consists of 7 items and is five-point Likert type [28]. The Turkish validity and reliability study of the scale was performed by Serefoglu et al. (2011). The lowest score that can be obtained from the scale is 7, and the highest is 35. If the score obtained is 31 and above, it is defined as “no premature ejaculation”; if it is between 26–30, as “mild premature ejaculation”; between 20–25, as “mild-moderate premature ejaculation”; between 14–19, as “moderate premature ejaculation”; and between 1–13, as “severe premature ejaculation”. The validity and reliability study of the scale, found the Cronbach alpha reliability coefficient to be 0.75 [4]. The internal consistency analysis for the scale of the study found the Cronbach alpha reliability coefficient to be 0.70.

### Applying the data collection tools

Data collection tools were applied by the researchers at Private LIV Hospital in Gaziantep and written permission was obtained from the hospital. Verbal and written consent forms were obtained by explaining the purpose of the study to the men, stating that the participation was completely voluntary and they could withdraw whenever they wanted. Data collection forms were completed individually by the participants.

#### Intervention

**Fig 2:**
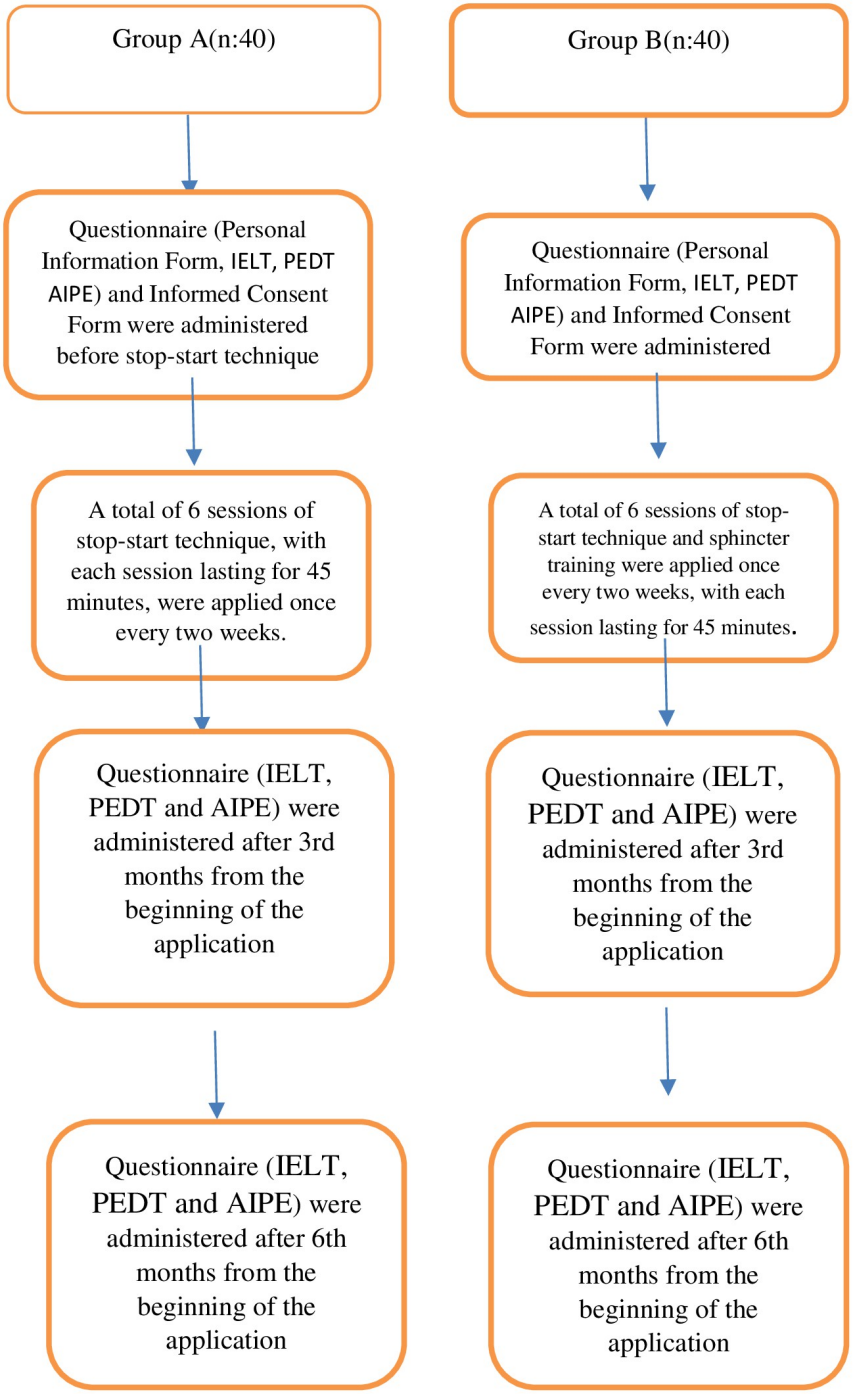
Flow plan of the research.

After researchers interviewed the men diagnosed with premature ejaculation according to the DSM-5 and ISSM criteria who applied to the urology outpatient clinic, they explained the purpose of the study and obtained written consent was from those who agreed to participate in the study. All of the patients in the study were men with lifelong premature ejaculation according to ISSM diagnostic criteria. The researchers administered Pre-tests (Personal Information Form, IELT, PEDT and AIPE) just before applying the behavioral treatment to the men in the study group. In the present study, the geometric mean of the IELT value in the last 2 weeks was calculated, and the IELT value was accepted. In the first interview with men with premature ejaculation problem, information about behavioral treatment was given, and treatment days and hours were determined. Afterwards, behavioral treatment consisting of a total of 6 sessions was applied once every two weeks for 45 minutes. In the treatment content, "stop-start technique" or "stop-start technique and sphincter control training" were used. Behavioral treatment interviews were conducted in the urology outpatient clinic of the hospital. Sessions lasted 3 months in total. The post-tests (IELT, F-IELT, PEDT, and AIPE) were administered at 3 months and 6 months from the beginning of the sessions. The interventions made are shown in the flow plan of the research (Fig 2).

#### Stop-start technique group (Group A)

For the first 3 sessions, sexual intercourse was prohibited. From the 4th session, sexual intercourse was allowed. The stop-start technique was explained with video and visuals. The stop-start technique was used once a day for 2 weeks. In this technique, the penis was stimulated until the feeling of ejaculation was felt, when the feeling of ejaculation was felt, the stimulation was stopped and the patient waited for a while, then the stimulation was continued again. It was repeated 5 times in total and the patient ejaculated voluntarily in the 6th repetition. The time from start to ejaculation was measured with a stopwatch. The ideal target time was 10–15 minutes. When the duration of the masturbation exercises was over 10 minutes, the next stage began; when it was under 10 minutes, the patients were told to continue the exercise for 2 more weeks (Table 1).

**Table 1:** Procedure of stop-start technique group.

Sessions	Procedure
***1st Session***	The patients were explained how to practice masturbation without having sexual fantasies.
***2nd Session***	The patients were told to masturbate by watching sexual fantasy or porn movies.
***3rd Session***	The patients were told that their partner should masturbate them
***4th Session***	It was explained that sexual intercourse should be performed once every two days for two weeks, with the man on the bottom and the woman on the top, in the cowboy position.
***5th Session***	It was explained that sexual intercourse should be performed once every two days for two weeks, with the woman lying on her back, and the man on the top, in the position where the man was active.
***6th Session***	Sexual intercourse was performed in different positions once every two days for two weeks. When the feeling of ejaculation came, the position was changed, 5 positions were changed, and the men were told that they could ejaculate voluntarily in the sixth position.

#### Stop-start technique and sphincter control training group (Group B)

For the first 3 sessions, sexual intercourse was prohibited. From the 4th session, sexual intercourse was allowed. A stop-start and sphincter control technique was used once a day for 2 weeks. Pelvic floor muscles, anal sphincter and external urethral sphincter were explained with video and visuals. Afterwards, kegel exercise and reverse kegel exercise were performed with biofeedback and awareness of these muscles was created. The patients were asked to do these exercises in sets of ten 3 times a day. It was explained that external urethral sphincter, anal sphincter, and pelvic floor muscles will be tried to be kept relaxed throughout masturbation. The patients were told to masturbate without having a sexual fantasy and to give a stimulation to the penis, and to stop when the feeling of ejaculation came, and then to take a deep breath and finally to exhale deeply. The men were asked to continue to stimulate the penis after five repetitions. They were told to repeat this sequence 5 times in total and to ejaculate voluntarily in the 6th repetition. The time from the start to ejaculation was to be calculated with a stopwatch. It was stated that our ideal target time was between 10–15 minutes. It was told that this would be done once a day for two weeks. When the duration of the masturbation exercises was over 10 minutes, the next stage began; when it was under 10 minutes, the patients were told to continue the exercise for 2 more weeks (Table 2).

**Table 2:** Procedure of stop-start technique and sphincter control training group.

Sessions	Procedure
***1st Session***	The patients were explained how to practice masturbation without having sexual fantasies.
***2nd Session***	They were told to masturbate while keeping the external urethral sphincter (EUS) and anal sphincter (AS) relaxed and while watching sexual fantasy or porn movies.
***3rd Session***	The patients were told that their partner should masturbate them
***4th Session***	It was explained that sexual intercourse should be performed once every two days for two weeks, with the man on the bottom and the woman on the top, in the cowboy position.
***5th Session***	It was explained that sexual intercourse should be performed once every two days for two weeks, with the woman lying on her back, and the man on the top, in the position where the man was active.
***6th Session***	Sexual intercourse was performed in different positions once every two days for two weeks. When the feeling of ejaculation came, the position was changed, 5 positions were changed, and the men were told that they could ejaculate voluntarily in the sixth position.

### Evaluation of data

After the data obtained from this research were coded by the researcher, it were transferred to the SPSS for Windows (Statistical Package for Social Sciences) computer program and the necessary analyzes were made in this program. In this study, “the stop-start technique” and “the stop-start technique and sphincter control training application” were independent variables, while “IELT”, “F-IELT”, “PEDT” and “AIPE” scores were dependent variables.

Shapiro-Wilk tests determined whether the data showed normal distribution. ANOVA was used for the intra-group comparison of the mean scores of the pre-test and post-test IELT, F-IELT, PEDT, and AIPE of the study group. Tukey test, one of the post-hoc tests, was applied to determine the means of the difference. Paired sample t-test was used for the intra-group comparison of the mean scores of the 3rd and 6th month IELT, PEDT, and AIPE of the study group. Student t-test was used to compare the mean scores of two independent groups and was considered significant at the p<0.05 level. In addition, mean, standard deviation, median, minimum and maximum values and percentages were used in the evaluation of the study findings.

## Results

A total of 80 participants, 40 patients in Group A and 40 patients in Group B, were included in the study. As can be seen in Table 1, both groups were equivalent in their demographic and clinical characteristics (p>0.05) (Table 3).

**Table 3:** Demographic and clinical characteristics of the patients.

Characteristics	Group A (n = 40)	Group B (n = 40)	p value	Cohen’s d
	x±sd	x±sd		
**Age (years)**	28.93±5.53	29.15±5.55	0.85	0.0009
**The presence of premature ejaculation (years)**	4.47±4.76	4.5±4.54	0.98	0.059
**IELT (seconds) (geometric mean)**	35.17±9.95	34.45±10.17	0.62	0.008
**PEDT (score)**	15.53±2.09	15.85±2.29	0.50	0.017
**AIPE (score)**	17.3±3.01	17.05±3.52	0.49	0.027

Student t test applied.

While the IELT mean score was 35.17±9.95 seconds before the application in Group A, it was 214.07(165–245) seconds (3.56 minutes) after three months from the beginning of the application and 215(175–242) seconds (3.58 minutes) after six months from the beginning of the application. It was determined that the IELT mean score after the application increased statistically in Group A compared to the pre-application (p = 0.001). In Group B, the IELT mean score before the application was 34.45±10.17 seconds, 537.52 (472–590) seconds (8.95 minutes) after three months from the beginning of the application and 553.7 (490–646) seconds (9.21 minutes) after six months from the beginning of the application. It was determined that the IELT mean score of Group B after the application increased significantly compared to the mean score of the pre-application (p = 0.001).

While the PEDT mean score before the application was 15.53(11–19) in Group A, it was 7.72(5–9) after three months from the beginning of the application and 7.65(5–9) after six months from the beginning of the application. It was determined that the PEDT mean score in Group A decreased statistically after the application (p = 0.001). While the PEDT mean value before the application was 15.85(12–20) in Group B, it was 5.75(2–8) after three months from the beginning of the application and 5.3(2–7) after six months from the beginning of the application. It was determined that the PEDT mean score of Group B decreased statistically after the application (p = 0.001).

While the AIPE mean score before the application was 17.3(12–20) in Group A, it was 27.07(22–31) after three months from the beginning of the application and 26.82(20–29) after six months from the beginning of the application. It was determined that the AIPE mean score in Group A increased statistically after the application (p = 0.001). While the AIPE mean score before the application was 17.05(12–20) in Group B, it was 31.52(31–33) after three months from the beginning of the application and 31.73(31–33) after six months from the beginning of the application. It was determined that the AIPE mean score of Group B increased statistically after the application (p = 0.001) (Table 4).

**Table 4:** Intra-group comparison of IELT, PEDT, and AIPE mean scores.

Clinical Results	Study Group	Mean before the application^(1)^ (min-max)	Mean after three months from the beginning of the application^(2)^ (min-max)	Mean after six months from the beginning of the application^(3)^ (min-max)	p value	Post-hoc
**IELT(geometric mean)**	**Group A**	35.17 (12–55)	214.07 (165–245)	215 (175–242)	0.001	1–2
						1–3
	**Group B**	34.45 (10–55)	537.52 (472–590)	553.7 (490–646)	0.001	1–2
						1–3
						2–3
**PEDT**	**Group A**	15.53 (11–19)	7.72 (5–9)	7.65 (5–9)	0.001	1–2
						1–3
	**Group B**	15.85 (12–20)	5.75 (2–8)	5.3 (2–7)	0.001	1–2
						1–3
						2–3
**AIPE**	**Group A**	17.3(12–20)	27.07(22–31)	26.82(20–29)	0.001	1–2
						1–3
						2–3
	**Group B**	17.05(12–20)	31.52(31–33)	31.73(31–33)	0.001	1–2
						1–3
						2–3

ANOVA test applied. Post-hoc: Tukey.

There was no statistically significant difference between the groups in terms of the IELT mean scores before the application (p = 0.62). It was determined that Group A’s IELT mean score was 214.07(165–245) seconds (3.5 minutes) and Group B’s IELT mean score was 537.52(472–590) seconds (8.9 minutes) after three months from the beginning of the application. The IELT mean score of Group B after three months from the beginning of the application showed a statistically higher increase compared to the IELT mean score of Group A and a low level of effect size was found between the two groups (p = 0.001, Cohen’s d = 0.011). It was determined that Group A’s IELT mean score was 215(175–242) seconds (3.58 minutes) and Group B’s IELT mean score was 553.7(490–646) seconds (9.2 minutes) after six months from the beginning of the application. Group B’s IELT mean score showed a statistically higher increase compared to Group A’s IELT mean score after six months from the beginning of the application and the difference in effect size between the two groups was clinically weak (p = 0.001, Cohen’s d = 0.025).

There was no statistically significant difference between the groups in terms of the PEDT mean scores before the application (p = 0.50). It was determined that the PEDT mean score of Group A was 7.72(5–9) and the PEDT mean score of Group B was 5.75(2–8) after three months from the beginning of the application. It was determined that the PEDT mean score of Group B decreased statistically more than the PEDT mean score of Group A after three months from the beginning of the application and a low level of effect size was found between the two groups (p = 0.001, Cohen’s d = 0.066). The PEDT mean score of Group A was determined to be 7.65(5–9) and the PEDT mean score of Group B was determined to be 5.3(2–7) after six months from the beginning of the application. It was found that the PEDT mean score of Group B decreased statistically more than the mean score of Group A after six months from the beginning of the application and a low level of effect size was found between the two groups (p = 0.001, Cohen’s d = 0.024).

There was no statistically significant difference between the groups in terms of the AIPE mean scores before the application (p = 0.494). The AIPE mean score of Group A was 27.07(20–29) and the AIPE mean score of Group B was 31.52(31–33) after three months from the beginning of the application. The AIPE mean score of Group B showed a statistically higher increase compared to the AIPE mean score of Group A after three months from the beginning of the application and the difference in effect size between the two groups was clinically weak (p = 0.001, Cohen’s d = 0.044). It was found that the AIPE mean score of Group A was 26.82(20–29) and the AIPE mean score of Group B was 31.73(31–33) after six months from the beginning of the application. The AIPE mean score of Group B showed a statistically higher increase compared to the AIPE mean score of Group A after six months from the beginning of the application and a low level of effect size was found between the two groups (p = 0.001, Cohen’s d = 0.048).

It was determined that Group A’s F-IELT mean score was 6.62±2.03 and Group B’s F-IELT mean score was 17.95±7.44 after six months from the beginning of the application. The F-IELT mean score of Group B after six months from the beginning of the application showed a statistically higher increase compared to the F-IELT mean score of Group A and the difference in effect size between the two groups was clinically weak (p = 0.001, Cohen’s d = 0.027) (Table 5).

**Table 5:** Intergroup comparison of IELT, PEDT, and AIPE mean scores.

Clinical Results	Mean Scores	Group A	Group B	p value	Cohen’s d
	**Mean before application (min-max)**	35.17 (12–55)	34.45 (10–55)	0.62	0.009
**IELT (geometric mean)**	**Mean after three months from the beginning of the application (min-max)**	214.07 (165–245)	537.52 (472–590)	0.001	0.011
	**Mean after six months from the beginning of the application (min-max)**	215 (175–242)	553.7 (490–646)	0.001	0.025
	**Mean before the application (min-max)**	15.53 (11–19)	15.85 (12–20)	0.50	0.017
**PEDT**	**Mean after three months from the beginning of the application (min-max)**	7.72 (5–9)	5.75 (2–8)	0.001	0.066
	**Mean after six months from the beginning of the application (min-max)**	7.65 (5–9)	5.3 (2–7)	0.001	0.024
**AIPE**	**Mean before the application (min-max)**	17.3 (12–20)	17.05 (12–20)	0.494	0.027
	**Mean after three months from the beginning of the application (min-max)**	27.07 (20–29)	31.52 (31–33)	0.001	0.044
	**Mean after six months from the beginning of the application (min-max)**	26.82 (20–29)	31.73 (31–33)	0.001	0.048
**F-IELT**	**Mean after six months from the beginning of the application (min-max)**	6.62±2.03	17.95±7.44	0.001	0.027

Student t test applied.

While the IELT mean score was 214.07 (165–245) seconds (3.56 minutes) in Group A after three months from the beginning of the application, it was 215(175–242) seconds (3.58 minutes) after six months from the beginning of the application. There was no statistically significant difference between the results of the IELT mean scores after three and six months from the beginning of the application in Group A (p = 0.214). In Group B, the IELT mean score after three months from the beginning of the application was 537.52(472–590) seconds (8.9 minutes), while the IELT mean score after six months from the beginning of the application was 553.7(490–646) seconds (9.2 minutes). A statistically significant difference was determined between the results of the IELT mean scores after three and six months from the beginning of the application in Group B and the difference in effect size in terms of time was clinically weak (p = 0.001, Cohen’s d = 0.009).

While the PEDT mean score was 7.72(5–9) after three months from the beginning of the application in Group A, it was 7.65(5–9) after six months from the beginning of the application, with no statistically significant difference (p = 0.323). While the PEDT mean score after three months from the beginning of the application was 5.75(2–8) in Group B, it was 5.3(2–7) after six months from the beginning of the application, with a statistically significant difference and a low level of effect size was found in terms of time (p = 0.001, Cohen’s d = 0.064).

While the AIPE mean score was 27.07(22–31) after three months from the beginning of the application in Group A, it was 26.82(20–29) after six months from the beginning of the application, with a statistically significant difference and a low level of effect size was found in terms of time (p = 0.003, Cohen’s d = 0.004). While the AIPE mean score after three months from the beginning of the application was 31.52(31–33) in Group B, it was 31.73(31–33) after six months from the beginning of the application, with a statistically significant difference and a low level of effect size was found in terms of time (p<0.019, Cohen’s d = 0.001) (Table 6).

**Table 6:** Intragroup comparisons of IELT, PEDT and AIPE mean scores three and six months after the application.

Clinical Results	Study Group	Mean after three months from the beginning of the application (min-max)	Mean after six months from the beginning of the application (min-max)	p value	Cohen’s d
**IELT (geometric mean)**	Group A	214.07 (165–245)	215 (175–242)	0.214	0.012
	Group B	537.52 (472–590)	553.7 (490–646)	0.001	0.009
**PEDT**	Group A	7.72 (5–9)	7.65 (5–9)	0.323	0.011
	Group B	5.75 (2–8)	5.3 (2–7)	0.001	0.064
**AIPE**	Group A	27.07 (22–31)	26.82 (20–29)	0.003	0.004
	Group B	31.52 (31–33)	31.73 (31–33)	0.019	0.001

Paired Sample t Test applied.

## Discussion

Premature ejaculation, which is one of the most common male sexual dysfunctions in the world and in our country, has many negative effects such as decreased sexual life quality, decreased self-esteem and decreased intimacy observed between couples, and increased interpersonal conflict [5, 29]. Considering all these negative effects, behavioral treatments and pelvic floor training to alleviate premature ejaculation are of great importance. Although there are many studies [16, 19, 23] examining the effects of behavioral therapy and pelvic floor exercises on men with premature ejaculation in the literature, no study has been found comparing the stop-start technique with the stop-start technique together with sphincter control training. This study is expected to contribute to the literature by investigating the stop-start technique, which is a current approach in the treatment of premature ejaculation, and comparing its results with the stop-start technique together with sphincter control training.

In this study, the mean age of the men in both groups was 29±5.51 and the problem of premature ejaculation was 4 years on average. The reasons the study subjects had not applied to the clinic earlier for treatment include the roles of men in society regarding sexuality, the fact that men think the problem will solve itself, the fact that men do not see premature ejaculation as a sexual dysfunction, and the fact that when men experience loss of self-confidence they do not share this problem with others.

The study observed that the stop-start technique applied to men with premature ejaculation significantly increased the IELT value compared to that in the pre-application period. This may be due to increased awareness of the ejaculation reflex. When we compared IELT values of the men in the stop-start technique group at three and six months, we found that there was no significant difference between them. The literature contains many studies examining the effects of the stop-start technique on men with premature ejaculation. Ventus et al. (2020) determined when men received the stop-start technique three times a week for a total of six weeks, the IELT value increased, but there was no difference between the results after three and six months from the beginning of the application [23]. At the same time, Cormio et al. (2015) determined that the IELT values of men with premature ejaculation who received behavioral treatment together with pharmacological treatment increased more compared to those who received pharmacological treatment alone [30]. Makwana and Patil (2012) stated the IELT value of 30 men receiving the stop-start technique increased [15]. These studies in the literature support our research findings. Our study observed that the IELT value of the men who received sphincter control training with the stop-start technique increased significantly compared to values in the pre-application period. Increased IELT values in men who received the stop-start technique together with sphincter control training may be due to increased awareness of and control over the ejaculation reflex. When we look at the comparison of IELT values at three and six months in men who underwent stop-start technique together with sphincter control training, a significant difference between them was found. The reason for this difference may be that the control over the ejaculation reflex strengthens over time. Rodríguez et al. (2019) determined that sphincter control training increased the IELT value [20]. However, no study in the literature was found applying the stop-start technique and sphincter control training together. It was determined that the IELT value increased more in men who received sphincter control training together with the stop-start technique compared to men who received the stop-start technique alone. This may be due to suppression of the ejaculation reflex along with relaxation of the external urethral sphincter and other pelvic floor muscles in addition to awareness in Group B, where only awareness was emphasized in Group A. In both groups, after six months from the beginning of the application, the F-IELT values increased significantly compared to the pre-application. However, the increase in Group B was higher than the increase in Group A. When we look at the studies in the literature, it was observed that the F-IELT value increased significantly compared to the pre-application level, similar to our study findings [17, 20].

This study observed that the stop-start technique significantly reduced the PEDT value compared to the pre-application. However, PEDT is a diagnostic tool. The use of the PEDT tool in comparing the effectiveness of treatment outcomes may have affected the results. This may be because the increase in the time spent in the vagina increases the self-confidence and sexual satisfaction of the individuals. Comparison of the PEDT values after three and six months from the beginning of the application presented that there was no significant difference between them. When we look at the studies in the literature, we see the studies on the effect of the stop-start technique on the PEDT value. For example, in the study of Pavone et al. (2017), it was observed that the PEDT value decreased after the application [16]. In Mantovani’s (2017) study on 18 patients, it was found that the PEDT value decreased in the post-test compared to the pre-test [31]. The PEDT value of the men who received sphincter control training with stop-start training decreased significantly compared to the pre-application. The reason why sphincter control training with the stop-start technique decreases the early PEDT value may be that the increased time in the vagina increases the self-confidence and sexual satisfaction of the individuals. Comparison of the PEDT value at three and six months in men who underwent sphincter control training with the stop-start technique shows a significant difference between them; this difference may be due to the fact that the control over the ejaculation reflex has strengthened with time, and sexual satisfaction has increased as psychological stress had diminished. Jiang et al. (2020) and Rodríguez et al. (2019) found that sphincter control reduces the PEDT value [19, 20]. However, no study was found in which stop-start technique together with sphincter control training were applied together. The reason why the PEDT value decreased more in men who received sphincter control training with the stop-start technique than men who received only the stop-start technique may be that the control over the ejaculation reflex was better learned in the group in which both techniques were applied, although the time spent in the vagina increased in both.

In the stop-start technique group, it was determined that the AIPE value after the application increased compared to before. The reason why the stop-start technique increases the AIPE value may be that the increased time spent in the vagina increases the sexual satisfaction of the person and his partner. The AIPE value increased significantly after the applying the stop-start technique together with the sphincter control training group. Comparing the AIPE value of the stop-start technique together with sphincter control training at three and six months shows a significant difference between them. The reason the AIPE value increased more in men who received sphincter control training with the stop-start technique compared to men who received only the stop-start technique may be the increased time spent in the vagina, as well as better control over the ejaculation reflex.

We think that the weak effect sizes of the intergroup and intragroup mean scores of the IELT, F-IELT, PEDT, and AIPE pre-treatment, the 3rd-month after the application, and the 6th-month after the application are due to the small number of samples and the similarity of the groups.

## Conclusions

Premature ejaculation continues to be a current health problem, as it includes complex situations that concern men and their partners. In the treatment of premature ejaculation, behavioral approaches merit attention in addition to pharmacological treatment. The stop-start technique together with sphincter control training is a treatment method that is cost-effective and easy to apply. However, the low clinical size levels suggest that this method should be studied on a larger sample. Therefore, we suggest expanding the application of non-pharmaceutical methods for the treatment of PE.

## Supporting information

S1 File (XLSX)

S2 File (XLSX)
